# Nitric Oxide Synthesis Is Increased in Cybrid Cells with m.3243A>G Mutation

**DOI:** 10.3390/ijms14010394

**Published:** 2012-12-24

**Authors:** Juliana Gamba, Luana T. Gamba, Gabriela S. Rodrigues, Beatriz H. Kiyomoto, Carlos T. Moraes, Celia H. Tengan

**Affiliations:** 1 Department of Neurology and Neurosurgery, Escola Paulista de Medicina, Universidade Federal de São Paulo, R. Pedro de Toledo, 781, São Paulo 04039-032, Brazil; E-Mails: juliana.gamba@unifesp.br (J.G.); luana_biologa@hotmail.com (L.T.G.); 1000gabi@gmail.com (G.S.R.); bhkiyomoto@unifesp.br (B.H.K.); 2 Department of Neurology and Cell Biology, Miller School of Medicine, University of Miami, Miami, FL 33101, USA; E-Mail: cmoraes@med.miami.edu

**Keywords:** mitochondria, nitric oxide, arginine, mitochondrial disease, mitochondrial DNA

## Abstract

Nitric oxide (NO) is a free radical and a signaling molecule in several pathways, produced by nitric oxide synthase (NOS) from the conversion of l-arginine to citrulline. Supplementation of l-arginine has been used to treat MELAS (mitochondrial encephalopathy with lactic acidosis and stroke like syndrome), a mitochondrial disease caused by the m.3243A>G mutation. Low levels of serum arginine and endothelium dysfunction have been reported in MELAS and this treatment may increase NO in endothelial cells and promote vasodilation, decreasing cerebral ischemia and strokes. Although clinical benefits have been reported, little is known about NO synthesis in MELAS. In this study we found that osteosarcoma derived cybrid cells with high levels of m.3243A>G had increased nitrite, an NO metabolite, and increased intracellular NO, demonstrated by an NO fluorescent probe (DAF-FM). Muscle vessels from patients with the same mutation had increased staining in NADPH diaphorase, suggestive of increased NOS. These results indicate increased production of NO in cells harboring the m.3243A>G, however no nitrated protein was detected by Western blotting. Further studies are necessary to clarify the exact mechanisms of l-arginine effect to determine the appropriate clinical use of this drug therapy.

## 1. Introduction

Nitric oxide (NO) is involved in the regulation of several pathways such as mitochondrial respiration, mitochondrial biogenesis and apoptosis, while it also can act as a free radical and cause oxidative damage. NO is produced by the isoenzyme nitric oxide synthase (NOS) which consumes l-arginine, producing NO and l-citrulline [1]. There are three isoforms of NOS: neuronal NOS (nNOS), endothelial NOS (eNOS) and inducible NOS (iNOS). The neuronal and endothelium forms are constitutive, but despite their denominations, they have also been identified in several other tissues. Both are activated by high levels of Ca^2+^ and requires the interaction between Ca^2+^ and calmodulin [1]. The iNOS is only activated during inflammatory or defense responses, is not dependent on Ca^2+^-calmodulin interaction and it is mainly expressed in macrophages [2]. In the brain, iNOS is induced in microglia and astrocytes by the presence of pathogens, brain damage or hypoxia [3].

Mitochondrial diseases are characterized by genetic defects leading to respiratory chain enzyme deficiency, with extremely variable clinical phenotypes, ranging from exercise intolerance to severe infantile encephaloneuromyopathies [4,5]. Until now, there is no effective pharmacological treatment for mitochondrial diseases [6]. One of the most devastating phenotypes of mitochondrial diseases is MELAS, mitochondrial encephalopathy with lactic acidosis and stroke like syndrome, which affects children and adults, with episodes that are very similar to strokes. MELAS is a very incapacitating disease because stroke-like episodes occur repeatedly, with progressive neurological deficits, including cortical blindness, convulsion and hemiparesis. In the majority of the cases, MELAS is caused by a mitochondrial DNA (mtDNA) mutation, m.3243A>G, in the *tRNA^Leu(UUR)^* gene, present in high levels in the affected tissues [7,8]. Muscle biopsy shows fibers with intense mitochondrial proliferation, called ragged red fibers (RRF), typically found in mitochondrial disorders. The same mitochondrial proliferation is also found in smooth muscle blood vessels in skeletal muscle biopsies, seen as vessels surrounded by intense blue on succinate dehydrogense (SDH) histochemistry, which are known as SSV, strong SDH-reactive blood vessels [9]. SDH and cytochrome *c* oxidase (COX) histochemical staining are largely used in the evaluation of mitochondrial function in muscle biopsies [6]. Because SDH is totally encoded by nuclear genes, it is not affected in mitochondrial diseases due to an mtDNA mutation. COX histochemistry is also very helpful, because it is frequently affected in patients with mtDNA mutations, with a pattern of focal COX deficiency, characterized by isolated and scattered muscle fibers with partial or total COX deficiency.

The pathogenesis of the stroke-like episode is still not clear. It could just be a manifestation of the cellular metabolic deficit or due to a deficiency in smooth muscle vascular relaxation, causing vasoconstriction and ischemia. Recently a Japanese group proposed a new therapy for MELAS based on reposition of l-arginine [10]. Their results are very promising when we consider clinical improvement because the patients had reduced frequency of stroke like episodes after short and long term administration of l-arginine [11]. However, the mechanism by which l-arginine acts is not understood. The basic concept for this treatment was the fact that previous studies demonstrated low levels of serum arginine [11] and endothelium dysfunction [9] in patients with MELAS. One of the functions of NO is to promote smooth muscle cell relaxation and vasodilation, so L-arginine would promote vasodilation in cerebral small vessels, which would improve neurological deficits in MELAS [12]. However little is known about basal NO synthesis in MELAS.

In this study, we aimed to evaluate NO synthesis in cells with the m.3243A>G mutation. We found that NO levels were increased in these cells and that NOS activity was not reduced in muscle vessels from patients with this mutation. Although increased levels of NO were found, no nitrated proteins were detected.

## 2. Results and Discussion

**Figure 1:**
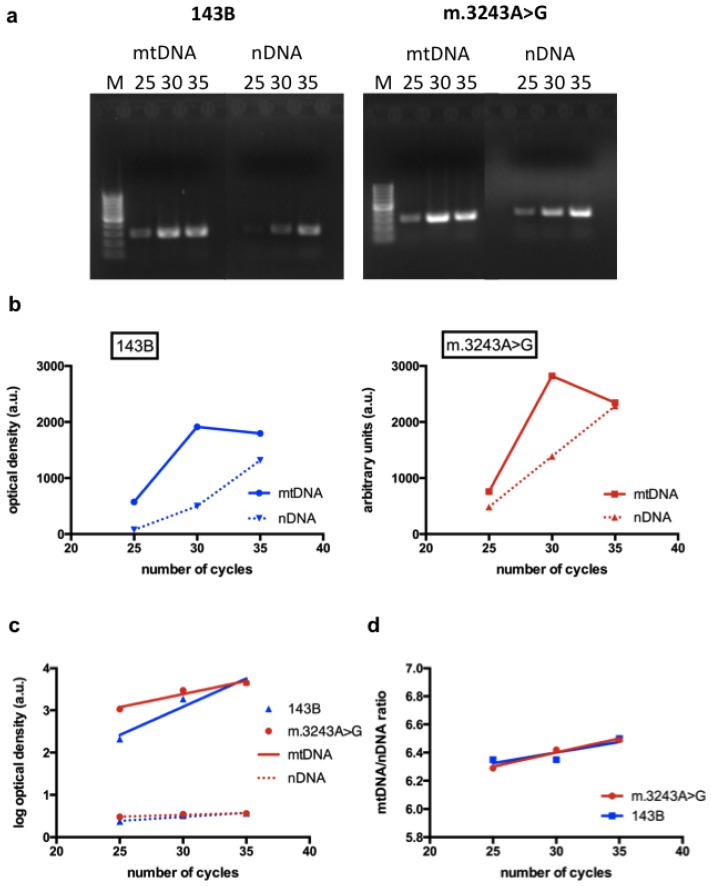
Evaluation of mtDNA content: (**a**) Polymerase chain reaction (PCR) products representative of an mtDNA segment and a nuclear gene (nuclear DNA, nDNA) are shown after 25, 30 and 35 cycles in a 2% agarose gel; (**b**) The intensity of the bands are expressed as optical density and are demonstrated in the graphs, showing the mtDNA bands (full line) and nDNA bands (dotted line). The graphs show that the interpolated curves obtained from the mtDNA have an exponential nature; (**c**) Using the log of optical densities, the relationship with number of cycles is linear for all parameters (mtDNA, nDNA, 143B and m.3243A>G), making the data comparable among different time points; (**d**) An mtDNA/nDNA ratio was obtained using the transformed results. The graph shows that the linear regressions obtained from 143B and m.3243A>G cells have similar slopes and intercepts, demonstrating that mtDNA content is similar in both cell lines. M = 100 bp ladder.

To study changes in NO synthesis related to MELAS mutation, we used transmitochondrial cybrid lines. Cybrid cells were derived from human osteosarcoma cell lines devoid of mtDNA, which were fused with enucleated cells containing mtDNA from patients [13]. They are very useful to study the effects of a specific mtDNA mutation, especially when using homoplasmic cells (containing 100% mutated mtDNA) in comparison with cells harboring 100% normal mtDNA (143B). The evaluation of the effect of a mutated mtDNA in tissue samples, such as muscle, is more difficult because the proportion of mutated molecules is variable in different cells. For this reason, we used cybrid cells with high level (93%) of the m.3243A>G mutation to verify the influence of this mutation on NO production. Although it would be ideal to use cybrid cells with 100% mutant mtDNA, the use of cells with a high level of mutant mtDNA, such as those used in this study, is also valid as far as they retain the phenotypic features caused by the mutation. We confirmed that cultured cells used in the experiments, retained their original features by checking the genotype and by confirming the decreased activities in complexes I, III and IV respiratory chain enzymes by spetrophotometric assays (Table 1). We also confirmed that mtDNA content was not altered in cybrid cells with the m.3243A>G compared to 143B cells (Figure 1).

**Figure 2:**
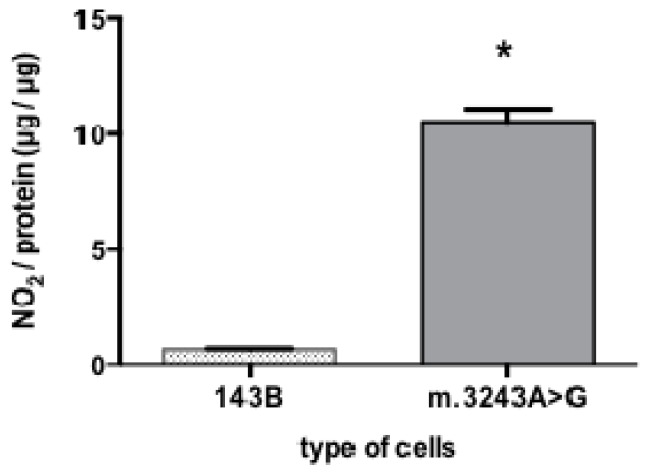
High nitrite content in cybrid cells with the m.3243A>G mutation. Cells with m.3243A>G showed a significant higher nitrite content (*p = 0.007) than 143B cells. Mann-Whitney test; number of samples =5; bars show means and standard deviation.

Because NO is a gaseous molecule with short half-life, direct NO detection and quantification is very difficult. Nitrite (NO_2_^−^) and nitrate (NO_3_^−^) are stable metabolites of NO and can be easily measured in body fluids, so the determination of nitrite is an indirect method but frequently used to evaluate NO production [14]. Our results showed that cells with m.3243A>G had a significant increase in the mean value of nitrite content (10.55 μg of nitrite per microgram protein) while 143B cells had only 0.68 μg of nitrite per μg protein (Figure 2).

**Figure 3:**
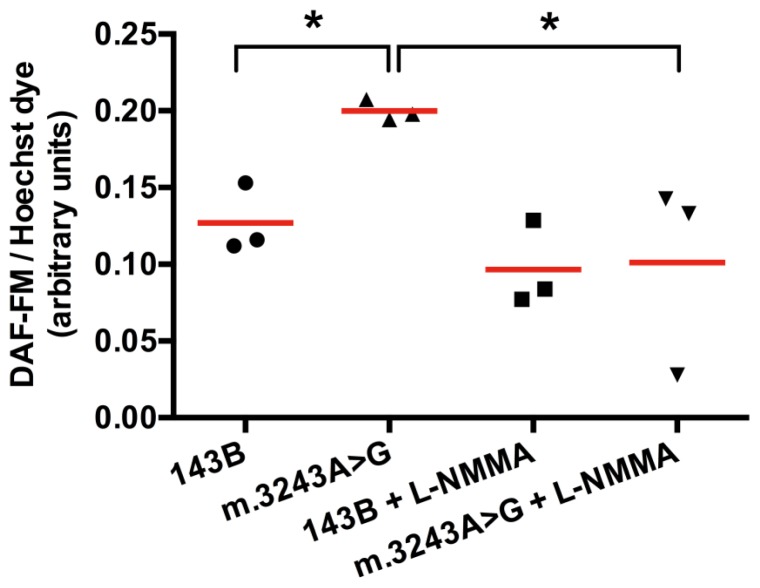
DAF-FM intracellular fluorescence is increased in cybrid cells with m.3243A>G mutation. Fluorescence obtained in cells with m.3243A>G was significantly increased when compared to the wild type (143B cell). Cells with mutated mtDNA had a significant decrease in fluorescence after treatment with l-NMMA, showing that NO detected by this probe was produced by NOS. *p = 0.05, Mann-Whitney test, number of samples =3, red horizontal lines = means.

To confirm that NO synthesis was increased, we used a fluorescent intracellular NO marker, DAF-FM (4-amino-5-methylamino-2′,7′-difluorfluorescein), which produces fluorescence when reacts with NO. DAF-FM fluorescence signal was corrected to the number of viable cells using Hoechst dye (nuclear marker) fluorescence as a reference. We obtained a significant higher fluorescence (mean = 0.20) in cells with mutated mtDNA when compared to 143B cells (mean = 0.13). After treatment with l-NMMA (N^G^-monomethyl-l-arginine acetate salt), a NOS inhibitor, the fluorescence was significantly reduced in cells with m.3243A>G mutation (mean = 0.10), while in 143B cells the difference was not significant with a mean value of 0.09 (Figure 3). After l-NMMA treatment, fluorescence intensity was similar in both cells, which we considered as a background. Higher concentrations of l-NMMA were not able to abolish this background (not shown). The increased signal in cells with mutated mtDNA was totally inhibited by l-NMMA, showing that this fluorescence was originated from NO produced by NOS.

**Figure 4:**
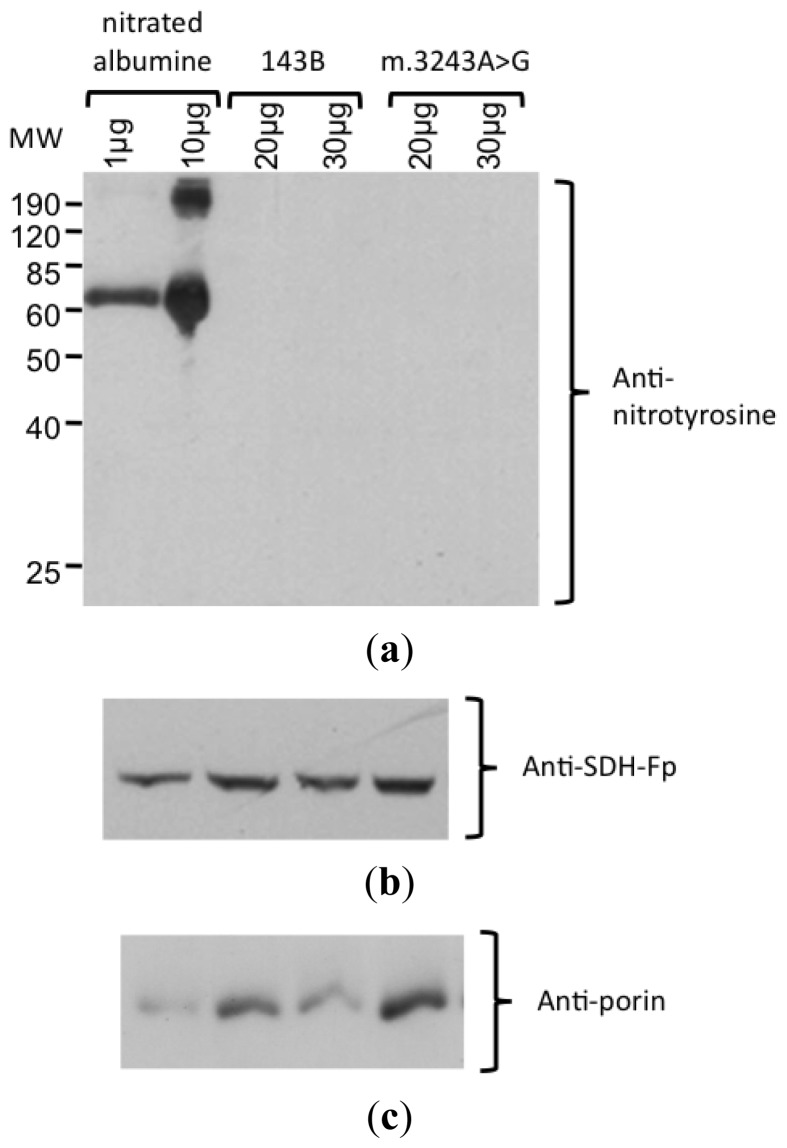
Detection of nitrated protein by Western blotting. (**a**) Incubation with anti-nitrotyrosine antibody showed the nitrated abumine (positive control), demonstrating that the antibody was specific. No nitrated protein was detected in samples from 143B and m.3243A>G cybrids, with 20 and 30 μg protein/lane. (**b)** Protein integrity was confirmed by the detection of the bands corresponding to SDH-Fp and (**c**) porin.

Because our results suggested higher levels of NO production in mutant cybrid cells, a Western blot analysis with an antibody against nitro-tyrosine was performed to evaluate the level of protein nitration. In this experiment, we could not detect any bands with this antibody using up to 200 μg of cell lysate protein (Figure 4). The specificity of the antibody was confirmed by the detection of nitrated albumine (positive control). The integrity of the proteins was confirmed by staining the membrane with Coomassie blue and obtaining the correct bands of other mitochondrial proteins, porin (mitochondrial membrane protein) and SDH-Fp (flavoprotein subunit of SDH).

Because MELAS is a mitochondrial disease with vascular involvement, we investigated NADPH (dihydronicotinamide adenine dinucleotide phosphate dehydrogenase) diaphorase (NADPHd) activity in small arterial vessels seen on muscle biopsies of patients with m.3243A>G mutation. NADPHd is a histochemical reaction used as an indirect method for detection of NOS activity. Muscle biopsies are the most available tissues to evaluate small vessels in these patients because most of the patients with a mitochondrial disease, have a muscle biopsy as part of the diagnostic investigation. We selected vessels from muscle biopsies of three patients with m.3243A>G mutation and compared to three controls (Table 2). The control samples were selected among diagnostic muscle biopsies and showed normal pattern of morphology and histochemistry, while clinical diagnosis were not related to mitochondrial diseases (Table 2).

**Figure 5:**
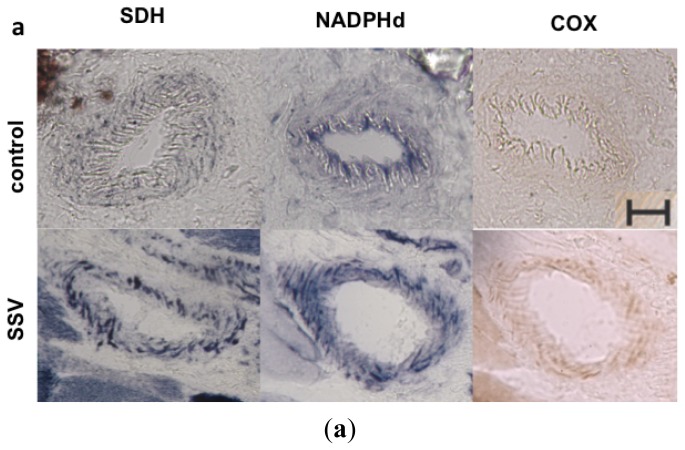

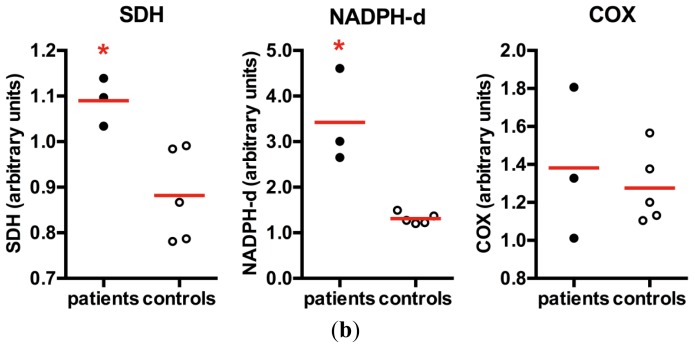
NADPHd in skeletal muscle vessels. (**a**) SSV and control vessel stained after histochemistry for SDH, NADPHd and COX; (**b**) Quantification of histochemistry staining in muscular vessels demonstrated that patients had a significant increase in SDH staining, confirming the mitochondrial proliferation in the vascular wall. NADPHd staining was also significantly increased in patients. However there was no increase in COX staining. *p = 0.036, Mann-Whitney test; red horizontal lines = mean; scale bar = 20 μm.

Quantitative analyses of histological staining of skeletal muscle vessels confirmed the increased mitochondrial content in patients with m.3243A>G mutation (mean = 1.09), with statistically significant increase in SDH staining in the vessels when compared to controls (mean = 0.88), typically found in SSV (Figure 5). A similar increase in NADPHd staining was also observed in SSV (mean in patients = 3.42, mean in controls = 1.31), suggesting that an increase in NOS activity or content of the enzyme. COX staining was not statistically significant (mean in patients = 1.38; mean in controls = 1.28) and did not increase proportionally to the SDH staining, suggesting a partial COX deficiency in skeletal muscle vessels.

The exact mechanism of stroke like episodes in MELAS is still unknown. There are mainly three hypotheses for the cause these episodes: (a) Angiopathy, leading to a decrease in arterial relaxation and hypoperfusion; (b) Cytopathy, causing a mitochondrial dysfunction and neural damage; (c) Neuronal hyperexcitability, due to imbalance between energy supply and demand [15]. l-arginine supplementation is a promising therapy for MELAS [9–11,16]. An improvement of neurological deficits in the acute phase and a decrease in the frequency of stroke like episodes with prolonged oral supplementation of l-arginine has been reported [9–11,16]. l-arginine treatment rationale is based mainly on the fact that patients with MELAS have decreased plasma levels of arginine [17,18], which is considered an NO precursor, leading to a deficiency in endothelium dependent vascular relaxation [18]. Using isotope infusion techniques, El-Hattab *et al.* found that whole body NO synthesis rate was low in patients with MELAS and increased after supplementation of arginine and citrulline [17]. This finding is interesting, but it is difficult to interpret considering that NOS are expressed in several tissues and the result is not tissue specific.

It is believed that NO deficiency may be caused by multiple factors: (a) Endothelial dysfunction; (b) NO sequestration by COX in COX-positive sites; (c) NO shunting into reactive nitrogen species formation; (d) Decreased availability of NO precursors (arginine and citrulline); (e) Increased asymmetrical dimethy-larginine (ADMA) concentration, an NOS endogenous inhibitor [19,20]. Vascular abnormalities are supported by observations of SSV and vasogenic edema in stroke-like episodes [18]. Because COX activity is present in SSV in patients with MELAS, it is hypothesized that COX hyperactivity may decrease regional NO concentration, leading to a segmental vasodilation defect especially in the segment of SSV regions in cerebral artery or arterioles [20]. Although SSV had COX activity in muscle vessels, we did not find increased activity in muscle vessels in patients in this study. NO synthesis may also be decreased due to increased levels of ADMA (asymmetrical dimetyl-arginine) and free radicals [18,19], which is supported by the finding of high levels of plasma concentration of ADMA in patients with MELAS [17].

Our results are not in agreement with the hypothesis of low NO synthesis, showing an opposite scenario. When analyzing NADPHd staining in SSV, we observed increased staining, suggestive of increase of NOS activity or expression. NADPHd is an indirect marker of NOS activity but with good correlation between NOS expression and NADPHd staining [21–23]. The specificity of this staining as an NOS activity indicator is a result of the use of Triton-X in the incubation medium, that enhances formazan generated by NOS related NADPHd [21]. Our results are supported by the finding of increased expression of eNOS in muscle blood vessels of patients with MELAS, while iNOS was not expressed in the same vessels [24]. Furthermore, in our previous work, we also found increased NOS activity in muscle of patients with m.3243A>G mutation, seen as increased NADPHd activity in the sarcoplasm of muscle fibers with mitochondrial proliferation and COX activity [23]. Although NO synthesis was increased in cybrids with m.3243A>G mutation, we could not detect any nitrated proteins in homogenates from cybrid cells by immunoblotting. Vattemi *et al.* were able to detect nitrated proteins in muscle blood vessels by immunostaining and in muscle homogenates by two dimensional immunoblot patients with MELAS [24]. The discrepancy of these results can be explained by the fact that we searched for nitrated proteins in cybrids homogenates, a different cell type, which could have lower levels of nitrated proteins, below the detection limit of this method. We have not checked for nitrated proteins in muscle blood vessels, but our finding of increased activity of NADPHd is in agreement with the presence of nitrated protein in this tissue, as NO synthesis is probably increased.

The comparison of our results with those from other studies showing decreased NO synthesis is difficult due to the diverse methods employed in each study [9,12,17]. Koga *et al.* [9,12] evaluated arginine and NO metabolites levels in plasma and urine of patients, which may not be good indicators of NO synthesis at the cellular level. Using an isotope infusion method, El Hattab *et al.* [17] also determined arginine, citrulline and NO metabolites in serum from patients, finding lower NO synthesis rate associated with reduced arginine and citrulline plasma concentration. Discrepant results are also found in other studies. Koga *et al.* [12] found reduced levels of NO metabolites in plasma and urine samples from patients in the acute phase while higher levels were observed in the inter-ictal phase. Vattemi *et al.* [24] could not find any differences in skeletal muscle NOS activity between controls and patients with mitochondrial disease by measuring (^3^H) l-arginine to (^3^H) l-citrulline conversion. However they found increased expression of eNOS in skeletal muscle homogenates of patients with mitochondrial diseases which supports our finding of increased NO synthesis in cybrid cells with the m.3243A>G mutation. Furthermore, in agreement with what was seen by Koga *et al.* [12], it is possible that we found higher values of NO synthesis due to the clinical phase, as the patients were not in acute phase of a stroke like episode.

Arginine and its metabolite products have several physiological roles, such as: ammonia detoxification, signal transduction, neuronal Na-K ATPase activity, neurotransmitter release, calcium homeostasis, maintenance of membrane potential and ion gradients, inhibition of cell proliferation and inhibition of NOS [25]. The regulation of arginine metabolism and mechanisms of its complex physiological roles still need clarification. Recently, Yoneda *et al.* [15] proposed that a second mechanism for the therapeutic effect of l-arginine is an acceleration of the tricarboxilic cycle (TCA) cycle. This hypothesis is based on the observation of a severe suppression of the TCA cycle metabolic rate in hearts of patients with mitochondrial cardiomyopathy, indicating a shift in energy production to the anaerobic pathway. l-arginine treatment was able to enhance the TCA cycle in these patients [26].

The basal levels of arginine in mutant cells and the direct effect of l-arginine supplementation on cybrid cells with the m.3243A>G mutation are interesting points that need further investigation. A recent study using cybrid cells derived from neuroblastoma cell line showed that cells with the m.3243A>G improved complex I activity with the addition of l-arginine in the culture medium [27]. In the same study, lower nitrite concentration was found in supernatant of mutant cells, suggesting that NO was decreased in these cells. Although we used the same method to determine nitrite content, our results are still difficult to compare, first, because there may be differences regarding the cell type as we used cybrids derived from osteosarcoma cell line, and second, because in our study we corrected the value of nitrite content to the total amount of protein in the plate, which was not done by Desquiret-Dumas *et al.* [27]. In another study, El-Hattab *et al.* [17] showed that citrulline supplementation had a greater efficacy in increasing NO production when compared to arginine supplementation, promoting a marked increase in the de novo arginine synthesis.

Because l-arginine therapy is not free of side effects [18,28,29], it is important to clarify the exact mechanisms of l-arginine improvement to determine the potential implications regarding benefits, dosage and side effects. Several issues need more clarification. We show that NO synthesis is increased in cells with the MELAS mutation, but we still do not know the exact role of this increase at the cellular or mitochondrial level. We can think in several possibilities. NO could have a protective role, acting in signaling for mitochondrial proliferation [30]; improving the blood flow and substrates supply to mitochondria or participating in redox signaling events [31]. On the other hand, increased NO can inhibit mitochondrial respiration and generate peroxynitrite, inducing nitrosative stress [32]. The NO roles on several signaling pathways are very complex and can be different depending on the cell type and intracellular location of NO synthesis. Further studies are still needed to clarify all those issues and discrepancies found in different studies but the knowledge of the basal levels of NO synthesis in mitochondrial deficient cells are crucial to a better understanding of l-arginine therapy.

l-arginine supplementation as a therapeutic approach is based on the assumption that there is an NO deficiency or that l-arginine will promote increase in NO synthesis and consequent vasodilation, improving the cerebral vascular blood supply. However, we show that NO synthesis is increased in cells with m.3243A>G mutation, which suggests another mechanism for the effects of l-arginine therapy.

## 3. Experimental Section

### 3.1. Cell Culture

Osteosarcoma derived cells with wild type mtDNA (143B) and the mutation m.3243A>G were used in this study. Cybrid cells were obtained from fibroblast cells of a patient with the 3243A>G mutation. Cells were grown at 37 °C with 5% CO_2_ in Dulbecco’s Modified Eagle’s Medium (DMEM) with high glucose (4,500 mg/L d-glucose) and 4 mM l-glutamine (Gibco BRL: Carlsbad, CA, USA), supplemented with 44 mM sodium bicarbonate (Sigma: Taufkierchen, Germany), 15% Fetal Bovine Serum (FBS), minimum essential media (MEM) vitamin solution, 10 mM MEM non-essential aminoacids solution, 100mM sodium pyruvate, penicillin (100 U/mL), streptomycin sulfate (100 μg/mL), fungizone (0.5 μg/mL) (all from Gibco BRL) and uridine (100 μg/mL) (Sigma).

### 3.2. Cell lines Characterization

#### 3.2.1. Genotyping

Detection of the 3243A>G mutation was performed by restriction fragment length polymorphism (RFLP) analysis with *Apa*I. We amplified a 293 bp fragment encompassing the 3243 position, using the following primers: forward (aggacaagagaaataaggcc) and reverse (tacgcaaaggccccaacgtt). Digestion with *Apa*I was performed at 37 °C for two hours, followed by separation in 1% agarose gel stained with ethidium bromide. The presence of the m.3243A>G mutation creates one site for *Apa*I, resulting in two bands (177 bp, 116 bp). Normal mtDNA does not have the restriction site and would not be cut. The percentage of mutated mtDNA was estimated by quantification of the intensity of the bands.

#### 3.2.2. Evaluation of mtDNA Content by Semi-Quantitative PCR

We performed the PCR amplification of two regions representing the mtDNA and nDNA. The mtDNA region was amplified using two primers (forward: 5′-aggacaagagaaataaggcc-3′; reverse: 5′-tacgcaaaggccccaacgtt-3′) encompassing the region between nucleotides 3130 and 3423). A segment containing exon 5 of the nuclear gene *NDUFV1* (a complex I subunit) was amplified using the primers: forward 5′-gctgggaaactcacaccttt-3′ and reverse 5′-caaagggagcctagccagat-3′. Total DNA was quantified using Hoechst dye as DNA fluorescent probe in the Versa Fluor fluorometer (Bio-Rad). PCR reaction was performed with 30 ng of total DNA, PCR buffer (Fermentas), 2.5 mM MgCl_2_, 20 nmol of each dNTP (deoxyribonucleotide triphosphates), 20 pmol of each primer and 2.5 units of Taq DNA polymerase (Fermentas). PCR conditions were: initial denaturing step at 94 °C for 2 min, followed by 35, 30 or 25 cycles with 94 °C (30 s) and 60 °C (30 s) and a final extension step at 72 °C for 10 min. PCR products were electrophoresed through a 2% agarose gel and photographed under UV light. The bands were quantitated by densitometry using the Image J 1.43r Software. The results were plotted against number of cycles and the ratio mtDNA/nDNA was obtained using the log of optical density of the bands obtained with 25, 30 and 35 cycles.

#### 3.2.3. Biochemical Enzyme Assays

##### 3.2.3.1. Isolation of Mitochondria

Biochemical enzyme assays were performed with mitochondrial enriched fractions obtained from 4 × 10^6^ cells [33], resuspended in 400 μL of mannitol buffer (225 mM mannitol, 75 mM sucrose, 10 mM Tris-HCl, 0.1 mM ethylenediamine tetraacetic acid, pH 7.2) [34] and homogenized in a hand-held Potter Elvehjem homogenizer in ice. The homogenates were centrifuged (600*g*, 4 °C, 10 min) and supernatant was transferred to another tube. A second step of homogenization of the same pellet was performed as above and then added to the first tube. Supernatants were centrifuged (15,000*g*, 4 °C, 10 min) to precipitate mitochondria. Pellets were resuspended in mannitol buffer and protein quantification was performed with the BCA Protein Assay kit (Pierce^®^). Adequacy of mitochondrial fraction was confirmed by the absence of a cytoplasmic enzyme activity, lactate dehydrogenase (EC 1.1.1.27) [35], and easy detection of other respiratory chain enzymes and citrate synthase (EC 4.1.3.7) activities [36].

##### 3.2.3.2. Complex I (NADH ubiquinone oxido-reductase, EC 1.6.5.3)

Immediately before the assay, mitochondria (20 μL, 1 μg/μL) were permeabilized with two cycles of freeze/thawing: Freezing in alcohol with dry ice and thawing at 37 °C. C-I assay [37] was performed in the Cary 50 spectrophotometer (Varian Inc.: Victoria, Australia) with readings during 6 min. We followed the oxidation of NADH to NAD+ (NADH extinction coefficient = 4.87) at 340 nm with 380 nm as the reference wavelength. The assay was performed at 37 °C, in an 1 mL cuvette with 25 mM K_2_HPO_4_ (pH 7.4), 5 mM MgCl_2_, 0.25% bovine serum albumin, 3.7 μM antimycin A, 2 mM KCN, 16% dimethyl sulfoxide, 40 mM reduced NADH. The reaction was started with the addition of the acceptor, ubiquinone 1, in the final concentration of 100 μM [37]. Extinction coefficient = 4.87 mM^−1^cm^−1^.

##### 3.2.3.3. Complex II (Succinate Decylubiquinone DCPIP Reductase, EC 1.10.2.2)

The assay was performed at 600 nm following the decrease in absorbance resulting from the reduction of 2,6-dichlorophenolindo-phenol (DCPIP). In 1 mL of medium (10 mM KH_2_PO_4_, pH 7.8, 2 mM EDTA, 1 mg/mL bovine serum albumin, BSA) we added 30 μg of mitochondria, 80 μM DCPIP, 4 μM rotenone, 0.2 mM ATP and 10 mM succinate. The mix was incubated for 10 min at 30 °C and the reaction started with the addition of 80 μM decylubiquinone and inhibited with 1 mM tenoiltrifluoracetone (TTFA) [38]. Extinction coefficient = 19.1 mM^−1^cm^−1^.

##### 3.2.3.4. Complex II + III (Succinate Cytochrome *c* Reductase, EC 1.3.5.1 + EC 1.10.2.2)

The assay was performed at 550 cm following the increase in absorbance resulting from the reduction of cytochrome *c*. In 1 mL of medium (25 mM KH_2_PO_4_, pH 7.2) we added 30 μg of mitochondria, 240 μM KCN, 4 μM rotenone and 20 mM succinate. The mix was incubated for 10 min at 30 °C and the reaction was started with the addition of 40 μM oxidized cytochrome *c* and inhibited with 1 mM tenoiltrifluoracetone [38]. Extinction coefficient = 19.1 mM^−1^cm^−1^.

##### 3.2.3.5. Complex IV (Cytochrome *c* Oxidase, EC 1.9.3.1)

The assay was performed at 550 nm following the decrease in absorbance resulting from the oxidation of reduced cytochrome *c*. In 1 mL of medium (10 mM KH_2_PO_4_, pH 6.5, 0.25 M sucrose, 1 mg/mL BSA) we added 30 μg of mitochondria and 10 μM reduced cytochrome c. We followed the reaction for 3 min and then added 2.5 mM lauryl maltoside. The reaction was inhibited by 240 μM KCN [38]. Extinction coefficient = 19.1 mM^−1^cm^−1^

##### 3.2.3.6. Citrate Synthase

Citrate synthase. The assay was performed at 412 nm following the reduction of 0.1mM 5,50-dithiobis (2-nitrobenzoic acid) in the presence of 30 μg of mitochondria, 0.2 mM acetyl-CoA in a medium with 10 mM Tris-HCl, pH 7.5 and 0.2% Triton X-100. The mix was incubated at 30 °C for 5 min and the reaction started with the addition of oxalacetic acid to a final concentration of 5mM [38]. Extinction coefficient = 13.6 mM^−1^cm^−1^.

### 3.3. Quantification of Nitrite

Nitrite content was determined in culture medium by a diazotation assay with the Griess reagent (Molecular Probes: Location, Country) and spectrophotometric detection [39]. Cells were grown until 90% confluence in 60 × 15 mm Petri dishes (TPP). After 72 h with no medium change, we incubated 300 μL of culture medium with equal volume of Griess reagent [40]. Nitrite content was determined according to a standard curve obtained from samples containing known concentrations of sodium nitrite and corrected by total protein content (μg of nitrite per μg protein). Cell pellets from each dish were homogenized in 10% sodium dodecil sulfate (SDS), 10 mM ethylenediamine tetraacetic acid, 6% glycerol, 10 mM ethylene glycol tetraacetic acid, 63 mM Tris (pH 6.8) and 50 mM dithiothreitol. Total protein was measured using the NanoOrange Reagent (Molecular Probes) according to manufacturer’s protocol.

### 3.4. Quantitative Analysis of Intracellular NO

DAF-FM diacetate (Molecular Probes), a cell membrane permeable probe, was used to detect intracellular NO. Once inside the cells, DAF-FM diacetate is deacetylated by intracellular esterases, becoming DAF-FM, which can be detected by fluorescent methods [41]. We plated 3 × 10^4^ cells in 12-well plates in DMEM high glucose, 10% FBS but with no phenol-red to avoid fluorescence interferences. The next day, the culture medium was replaced by DMEM high glucose without phenol-red and without FBS. After 24 h, with 90% confluence, cells were washed with DMEM without phenol-red or FBS and incubated with 5 μM DAF-FM diacetate at 37 °C with 5% CO_2_ for 1 h. Control samples were incubated with culture medium in the absence of DAF-FM diacetate. After incubation, cells were maintained for an additional 30 min in fresh medium to allow complete de-esterification of the intracellular diacetate. Then, cells were removed with TrypLE Express (Gibco BRL) and cell pellets were ressuspended in 1 mL DMEM high glucose without phenol-red or FBS. The fluorescence at 495 nm (excitation) and 515 nm (emission) was determined in a fluorometer (Bio-Rad Versa Fluor™ Fluorometer) with 1 mL cuvette. Cells were also incubated with 1 μg/mL Hoechst dye-33258 (Molecular Probes) for additional 15 min at room temperature and fluorescence was determined at 360 nm (excitation) and 460 nm (emission). Specificity of DAF-FM, as an NO probe, was checked by treating the cells with 1 mM l-NMMA (NOS inhibitor, Sigma) for 24 h.

### 3.5. Detection of Nitrate Protein by Western Blotting

We looked for nitrated proteins by performing a Western blotting analysis using an antibody against nitrotyrosine. Cell pellets were homogenized and 20–200 μg of total protein was run through a 12% denaturing polyacrylamide gel and electro-blotted to a 0.2 μm Sequi-Blot PVDF (polyvinylidene difluoride) membrane. After blocking with 5% milk in Tris-buffered saline Tween™-20 (TBS-T, 20 mM Tris, 137 mM sodium chloride pH 7.6, 0.1% Tween™-20), the membrane was incubated with the primary antibodies (anti-nitrotyrosine, anti-SDH-Fp, and anti-porin) diluted in 1% milk in TBS-T, for 1 h at room temperature. Incubation with the secondary antibody (horseradish peroxidase conjugated antibody anti-rabbit IgG and anti-mouse IgG, 1:10,000 dilution) was performed for 1 h at room temperature and the bands were obtained through chemiluminescent detection with ECL System (Amersham Biosciences).

A positive control was obtained by overnight incubation of 6 mg/mL BSA (Sigma) in a solution with 10 mM sodium nitrite, 20 mM sodium acetate pH 5.6, 9 μM cupper chloride, 0.3% hydrogen peroxide at room temperature [42].

In order to verify the integrity of the proteins, the membrane was stained with 0.1% amido black in 5% methanol and 10% acetic acid for 5 min. Primary antibody concentrations were: 1 μg/mL (anti-nitrotyrosine), 0.5 μg/mL (anti-SDH-Fp) and 2 μg/mL (anti-porin), all from Molecular Probes.

### 3.6. Muscle Histochemistry

We selected muscle biopsies from 3 patients with the m.3243A>G showing mitochondrial proliferation in skeletal muscle small vessels (SSV). Three muscle biopsies with no abnormalities and from patients with no clinical evidence of mitochondrial or metabolic myopathy were used as controls. We specifically analyzed arterial vessels seen on muscle biopsies after histochemical reactions for COX, SDH and NADPHd. Specificity of this staining was studied previously and certified by the co-localization of NOS expression and NADPHd staining [21,22].

### 3.7. Quantification of NOS Activity in Muscle Vessels

Microscope images containing muscle vessels were obtained through a 20× objective, with the same luminosity and in serial sections with NADPHd, COX and SDH histochemistry. All images were placed in one archive, with no alteration in size and as JPEG format (150 pixels/inch). The analyses were performed using Image J software (1.43r); RGB images were converted to 8 bits color, with scale from 0 to 256. With the Brush tool, we selected the whole vessel wall and performed the measurements. Measurements were normalized using the measure obtained in one type II fiber of the same section, in order to allow comparability of all samples.

### 3.8. Statistical Analysis

Statistical analyses were performed using Prism 6 for MacOS X (GraphPad Software Inc.: La Jolla, CA, USA). Comparisons were done with the Mann Whitney test. Statistical significance was set to *p* ≤ 0.05.

## 4. Conclusions

This study shows that NO production is increased in cybrid cells with the mutation m.3243A>G but did not show any evidence of nitration of proteins. Muscle vessels had increased NADPHd activity suggesting that NOS activity is high in these cells. These results indicate that l-arginine therapy may be acting by other mode of action that does not involve a vasodilation defect due to decreased NO.

Further studies must be performed to clarify the exact mechanisms of l-arginine effect to determine the appropriate clinical use of this drug therapy.

## Supplementary Information



## Figures and Tables

**Table 1:** Respiratory chain enzyme activities by spectrophotometry.

Cell type	C-I (nmol/mg/min)	C-II (nmol/mg/min)	C-II+III (nmol/mg/min)	C-IV (nmol/mg/min)	CS (nmol/mg/min)
143B	158.11	109.68	70.68	78.53	16.54
m.3243A>G	18.48	112.82	31.41	29.84	32.35

Notes: C-I = complex I; C-II = complex II; C-II+III = complex II + III; C-IV = complex IV; CS = citrate synthase.

**Table 2:** Patients used for the study of muscle small vessels.

Patient	Age at biopsy	Clinical diagnosis	Muscle biopsy	m.A3243A>G (%)	Number of vessels
1	45	exercise intolerance, myopathy	RRF, COX+	56	1
2	19	MELAS	RRF, COX+	67	1
3	4	MELAS	RRF, COX+	46	1
4	31	fatigue	normal	absent	3
5	23	fatigue	normal	absent	1
6	43	non-specific dermatitis	normal	absent	1
