# Supplementary material for: Nitric Oxide Synthesis Is Increased in Cybrid Cells with m.3243A>G Mutation
Source: Int J Mol Sci. 2012 Dec 24;14(1):394–410. doi: 10.3390/ijms14010394 (PMC3565270; doi:10.3390/ijms14010394)
Supplement: Supplementary file 1 [file ijms-14-00394-s001.pdf]

Supplementary Information

Figure S1. Quantification (**a**) of SDH-FP and porin bands obtained after Western blotting (**b**) using 20µg of total protein. The figure shows a slight increase (1.2 fold) of SDH-Fp band and a 1.6 fold increase in porin band in cybrid cells with the m.3243A>G mutation.

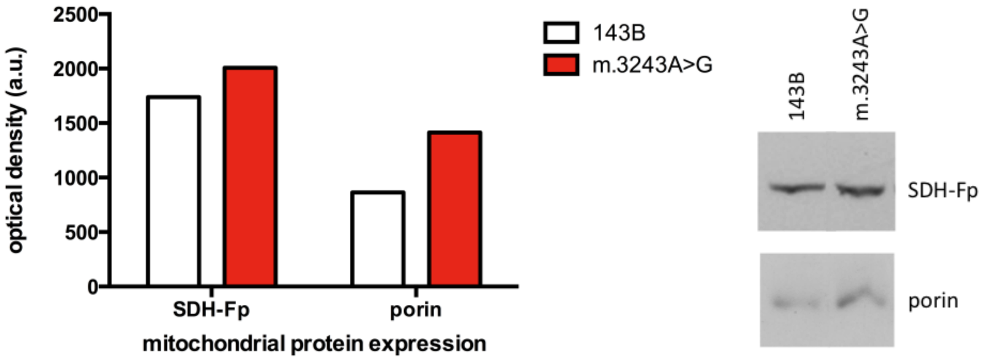

(**a**) (**b**)

© 2013 by the authors; licensee MDPI, Basel, Switzerland. This article is an open access article distributed under the terms and conditions of the Creative Commons Attribution license (http://creativecommons.org/licenses/by/3.0/).
